# Anti-VEGF Agents for the Treatment of Pigment Epithelial Detachments Associated with Neovascular Age-related Macular Degeneration: An Evidence-based Approach

**Published:** 2015

**Authors:** Georgios D. Panos, Zisis Gatzioufas

**Affiliations:** 1 Department of Ophthalmology, Geneva University Hospitals, Faculty of Medicine, University of Geneva, Geneva, Switzerland; 2 Moorfields Eye Hospital, London, UK

**Keywords:** Anti-VEGF Agents, Pigment Epithelial Detachments, Age-related Macular Degeneration

Age-related macular degeneration (AMD) is one of the main causes of irreversible blindness in industrialized countries (1). It has been classified into a non-exudative or dry form and a neovascular or wet form. Visual prognosis is worst in patients affected by the neovascular form (2).

Pegaptanib (Eyetech Pharmaceuticals and Pfizer), which was approved by the FDA at the end of 2004, was the first anti-VEGF therapy for neovascular AMD (3). After approval of bevacizumab (Genentech/ Roche) for cancer therapy and in view of the suspected role of VEGF in wet AMD, systemic intravenous and, later, intravitreal bevacizumab began to be administered off-label to treat neovascular AMD (4). Ranibizumab (Genentech/ Novartis), a humanized monoclonal anti-Fab antibody, engineered to bind with high affinity to VEGF-A and inhibit all of its biologically active isoforms, was found to be an effective therapeutic agent for neovascular AMD. This was proven in two pivotal trials: the Minimally Classic/Occult Trial of the Anti-VEGF Antibody Ranibizumab in the Treatment of Neovascular Age-Related Macular Degeneration (MARINA) and the Anti-VEGF Antibody for the Treatment of Predominantly Classic Choroidal Neovascularization in Age-Related Macular Degeneration (ANCHOR) (5). Based on this evidence, ranibizumab was approved by the FDA in 2006 as therapy for the treatment of wet AMD (6).

Most AMD clinical trials of anti-VEGF agents, such as ANCHOR and MARINA, have excluded eyes of the patients with large pigment epithelial detachments (PEDs) or, if such eyes were included, further analysis of the PED response to treatment was not performed. Therefore, the management of PEDs in patients with AMD is not clear and the usage of anti-VEGF in these cases remains controversial.

Lommatzsch et al. retrospectively studied 328 patients with serous PEDs associated with wet AMD. They observed that bevacizumab or ranibizumab are significantly more efficient in improving vision and retinal thickness than pegaptanib alone or in combination treatment with intravitreal triamcinolone acetonide and photodynamic therapy (7). In contrast, Introini et al., in a retrospective study of 132 eyes, concluded that there is no effective therapy for PED secondary to AMD and that anti-VEGF treatment could achieve only stabilization of the disease with the high risk of RPE tear (8). On the other hand, Arora and McKibbin analyzed eyes of 19 patients with vascularized and avascular PED, reporting that moderate visual loss was avoided in 95% of their patients and that 25% had a gain of 15 or more ETDRS letters, concluding that ranibuzumab is effective for both vascularized and avascular PED (9). In a prospective, comparative study, consisted of 15 patients, Arias proved that pegaptanib and bevacizumab were both an effective and safe therapeutic option for PEDs secondary to wet AMD (10).

Inoue et al. were the first to evaluate the outcome of Ranibizumab treatment in patients with neovascular AMD according to the type of PED (11). This prospective study consisted of 56 patients (11 serous PED, 28 fibrovascular PED, 7-mixed PED, and 10-haemorrhagic PED). Patients, treated with intravitreal ranibizumab according to the PrONTO study protocol, were followed-up over a period of 12 months after the initial injection. Results demonstrated that ranibizumab was effective in stabilizing or improving vision in patients with PED, but with better results in patients with serous PED (11). Our Group observed last year the favorable effect of ranibizumab on serous and fibrovascular PEDs associated with neovascular AMD (12). According to our study, ranibizumab showed effectiveness in improving vision and macular anatomy in patients with fibrovascular or serous PED secondary to wet AMD, however the anatomical response of the PED to the treatment may not be correlated directly with the visual outcome.

Two recent studies of the effect of aflibercept (Bayer Healthcare/ Regeneron Pharmaceuticals) on PEDs secondary to neovascular AMD have had promising outcomes. They concluded that aflibercept might be an effective alternative option for serous PED in neovascular AMD patients after bevacizumab and ranibizumab have previously failed (13) and that aflibercept may stabilize the visual acuity and improve the macular anatomy during the first 6 months of the treatment (14).

At present, anti-VEGF therapy is widely used in ophthalmology. Diseases such as neovascular AMD, macular edema secondary to retinal vein occlusion, diabetic macular edema, choroidal neovascularization secondary to causes other than AMD, and corneal neovascularization are treated with anti-VEGF drugs (5, 15-17). Regarding the efficiency of these drugs in the treatment of PEDs secondary to neovascular AMD, there is increasing evidence that they represent an effective and safe treatment approach that can stabilize or even improve the vision of patients and the macular anatomy. Larger studies in this field, especially about the efficacy of the newer anti-VEGF drug, aflibercept, are expected with great interest.
